# Large-scale annotation dataset for fetal head biometry in ultrasound images

**DOI:** 10.1016/j.dib.2023.109708

**Published:** 2023-10-20

**Authors:** Mahmood Alzubaidi, Marco Agus, Michel Makhlouf, Fatima Anver, Khalid Alyafei, Mowafa Househ

**Affiliations:** a College of Science and Engineering, Hamad Bin Khalifa University, Doha 34110, Qatar; b Sidra Medicine, Doha 34110, Qatar; c College of Health Sciences, University of Doha for Science and Technology, Doha, 24449, Qatar

**Keywords:** Fetal ultrasound imaging, Computer vision, Data annotation, Medical imaging

## Abstract

This dataset features a collection of 3832 high-resolution ultrasound images, each with dimensions of 959×661 pixels, focused on Fetal heads. The images highlight specific anatomical regions: the brain, cavum septum pellucidum (CSP), and lateral ventricles (LV). The dataset was assembled under the Creative Commons Attribution 4.0 International license, using previously anonymized and de-identified images to maintain ethical standards. Each image is complemented by a CSV file detailing pixel size in millimeters (mm). For enhanced compatibility and usability, the dataset is available in 11 universally accepted formats, including Cityscapes, YOLO, CVAT, Datumaro, COCO, TFRecord, PASCAL, LabelMe, Segmentation mask, OpenImage, and ICDAR. This broad range of formats ensures adaptability for various computer vision tasks, such as classification, segmentation, and object detection. It is also compatible with multiple medical imaging software and deep learning frameworks. The reliability of the annotations is verified through a two-step validation process involving a Senior Attending Physician and a Radiologic Technologist. The Intraclass Correlation Coefficients (ICC) and Jaccard similarity indices (JS) are utilized to quantify inter-rater agreement. The dataset exhibits high annotation reliability, with ICC values averaging at 0.859 and 0.889, and JS values at 0.855 and 0.857 in two iterative rounds of annotation. This dataset is designed to be an invaluable resource for ongoing and future research projects in medical imaging and computer vision. It is particularly suited for applications in prenatal diagnostics, clinical diagnosis, and computer-assisted interventions. Its detailed annotations, broad compatibility, and ethical compliance make it a highly reusable and adaptable tool for the development of algorithms aimed at improving maternal and Fetal health.

Specifications Table

**Table:** 

Subject	Computer Vision and Pattern Recognition.
Specific subject area	Ultrasound Fetal head dataset for computer vision tasks in prenatal diagnostics.
Data format	Raw, Analyzed
Type of data	Table, Image
Data collection	The dataset consists of 3832 high-resolution ultrasound images of Fetal heads, specifically focusing on the brain, cavum septum pellucidum (CSP), and lateral ventricles (LV). The images are 959×661 pixels and were obtained from public source under the Creative Commons Attribution 4.0 International license. They are anonymized and de-identified for ethical compliance. The annotations were validated through a two-step process involving a Senior Attending Physician and a Radiologic Technologist, using Intraclass Correlation Coefficients (ICC) and Jaccard similarity indices (JS) for reliability assessment.
Data source location	The data were collected from two publicly available sources under the Creative Commons Attribution 4.0 International license. Source 1: https://zenodo.org/record/3904280 Source 2: https://zenodo.org/record/1322001
Data accessibility	Repository name: Zenodo Data identification number: DOI10.5281/zenodo.8265464 Direct URL to data: https://doi.org/10.5281/zenodo.8265464 Instructions for accessing these data: The dataset is publicly available and can be downloaded directly from the Zenodo repository using the provided DOI.

## 1 Value of the Data

• Rich Annotation and Validation: This dataset stands out for its meticulous annotation and two-step validation process involving medical experts. It offers a reliable foundation for machine learning models in medical imaging, particularly in prenatal diagnostics.
• Versatility in Formats: The dataset's availability in 11 universally accepted formats ensures its compatibility with a variety of software tools and deep learning frameworks. This makes it highly adaptable for researchers from different domains.
• High-Resolution and Comprehensive: With 3832 high-resolution ultrasound images focusing on key areas like the Fetal brain, CSP, and LV, the dataset is one of the most comprehensive of its kind, making it a valuable resource for robust algorithm training and validation.
• Reusable and Customizable: The dataset does not include predefined training and testing splits, allowing other researchers to tailor the data partitioning according to the specific requirements of their projects. This facilitates reuse in various medical and computational studies.
• Broad Application Scope: While the dataset is highly specialized, its wide range of applications from prenatal diagnostics to computer-assisted medical interventions makes it valuable to researchers, clinicians, and data scientists alike.

## 2 Data Description

**Fig. 1:**
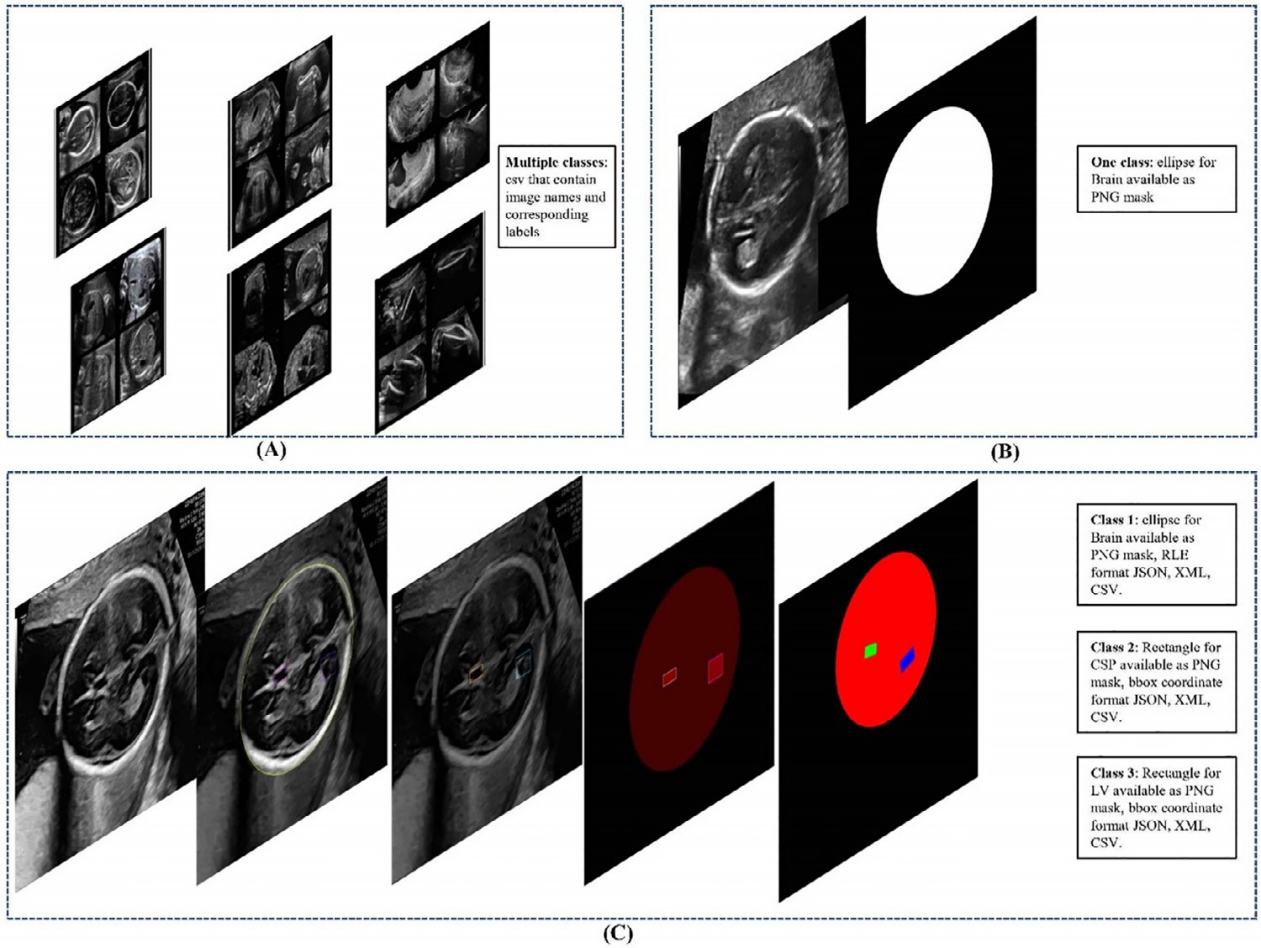
Comparison between existing Fetal ultrasound datasets and our new dataset. Panel (A) shows the Fetal plane classification dataset, panel (B) shows the Fetal head segmentation dataset, and panel (C) shows our dataset, which includes various formatting options for each class.

This artcle describe a dataset that we reproduced from two publicly available dataset as seen in Table 1. Dataset A [1] includes 12,400 ultrasound images, specifically designed to classify Fetal planes such as the brain, abdomen, and thorax. Dataset B [2], comprising 999 images of the Fetal head with the corresponding masks, was explicitly created for the segmentation of the Fetal head. To bridge this gap, we used two existing datasets to assemble a novel dataset tailored to address issues related to Fetal head ultrasound images. Our dataset is available for download at zenodo repstiry [3]. (Fig. 1) illustrated various formats that avaialble for our dataset C and compare it with previouse exising dataset A and B.

**Table 1:** An overview of existing Fetal head ultrasound image datasets including computer vision task, and number of class.

Dataset ID	Dataset	Computer vision task	Number of classes	Format	Size
A	Fetal_Plane_DB	Image Classification	9	PNG images with classes	12400
B	Fetal_head_HC18_Grand	Image segmentation	1	PNG images with corresponding masks	999
C	Our dataset	Classification, Segmentation, and object detection	3	PNG images with the following format: CityScapes, Datumaro, COCO, CVAT, ImageNet, LabelMe, OpenImage, PASCAL, Segmentation masks, TFRecord, YOLO	3832

**Fig. 2:**
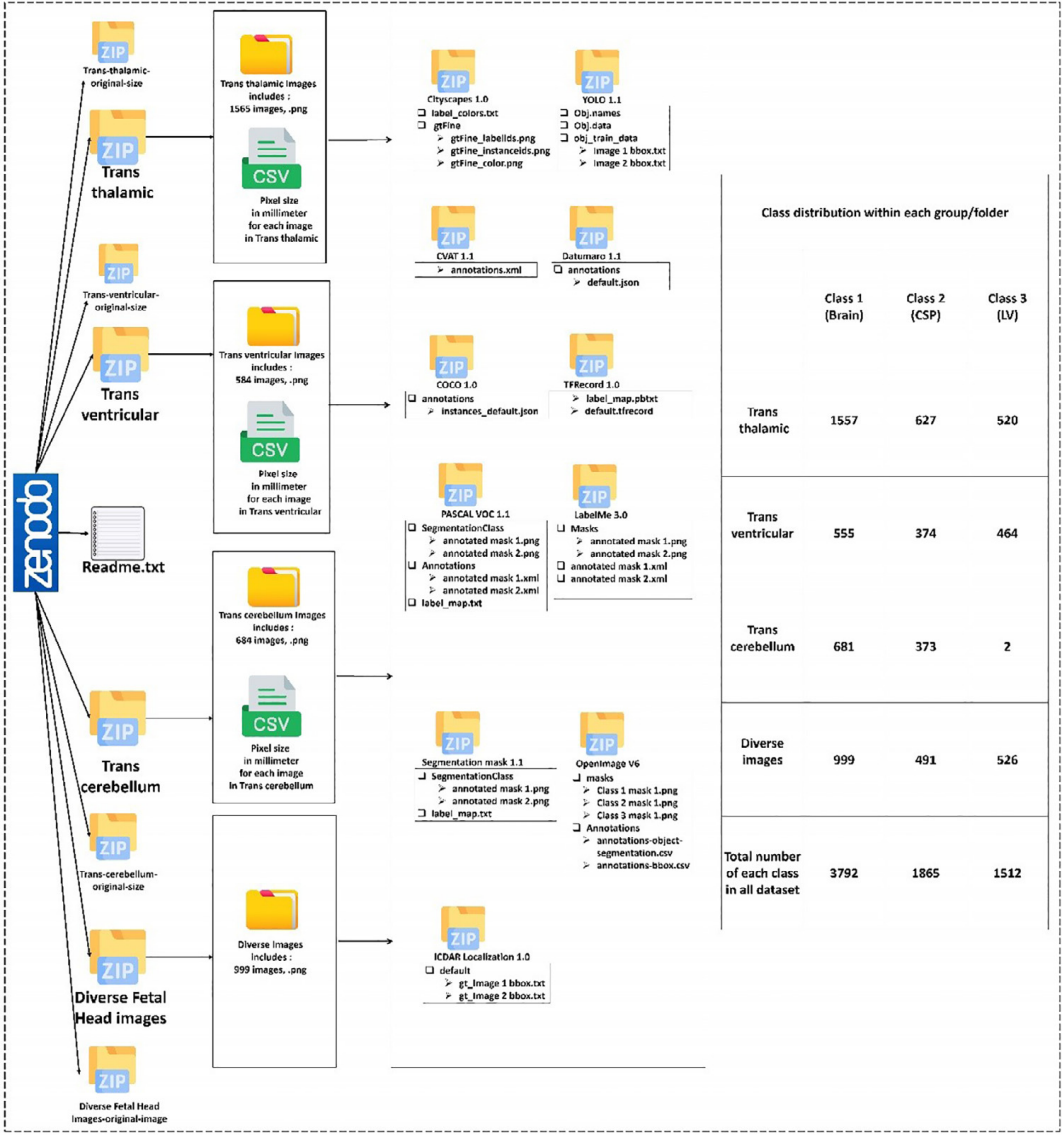
Dataset structure visualized in four main zip folders by Fetal plane, including sub-zip folders for 11 data formats, and table indicating class instance counts (Brain: 3794, CSP: 1865, LV: 1512).

The dataset currently released consists of 3832 high-resolution head ultrasound images, each with dimensions of 959 × 661 pixels. The dataset does not include predefined training and testing splits, allowing users to customize their partitioning based on the specific requirements of their projects. As depicted in (Fig. 2) the folder structure of the dataset includes subfolders and files designed for computer vision tasks such as classification, segmentation, and object detection. The dataset is thoughtfully organized into four main zip folders, corresponding to specific groups of the Fetal plane (trans thalamic, trans ventricular, trans cerebellum, and various images of the Fetal head). Additionally, four zip folders are provided that contain the original images for each group before resizing. These original images serve as a valuable reference, enabling researchers to adjust or resize masks/labels as needed. A readme.txt file is also included to offer a concise summary of the dataset.

Each main zip folder consists of two key components: a folder with the resized images and a CSV file specifying the pixel sizes in millimeters (mm) for each image. Within each of the four main zip folders, 11 sub-zip folders are present, each representing one of the 11 different formats supported by the dataset, ensuring adaptability to various annotation requirements.

Fig. 2 visually represents the quantity of instances for each class within each group of the Fetal plane, with the brain class comprising 3794 instances, the CSP class containing 1865 instances, and the LV class totaling 1512 instances. The well-structured organization of this dataset ensures easy access to both standardized and original images, meeting a broad spectrum of research needs and providing significant flexibility in utilizing the dataset. The 11 formats supported in our dataset are Cityscapes [4], You Only Look Once (YOLO) [5], CVAT, Datumaro, The Common Objects in Context (COCO) [6], TFRecord, PASCAL [7], LabelMe [8], Segmentation mask [7], OpenImage, and ICDAR [9].

## 3 Experimental Design, Materials and Methods

**Fig. 3:**
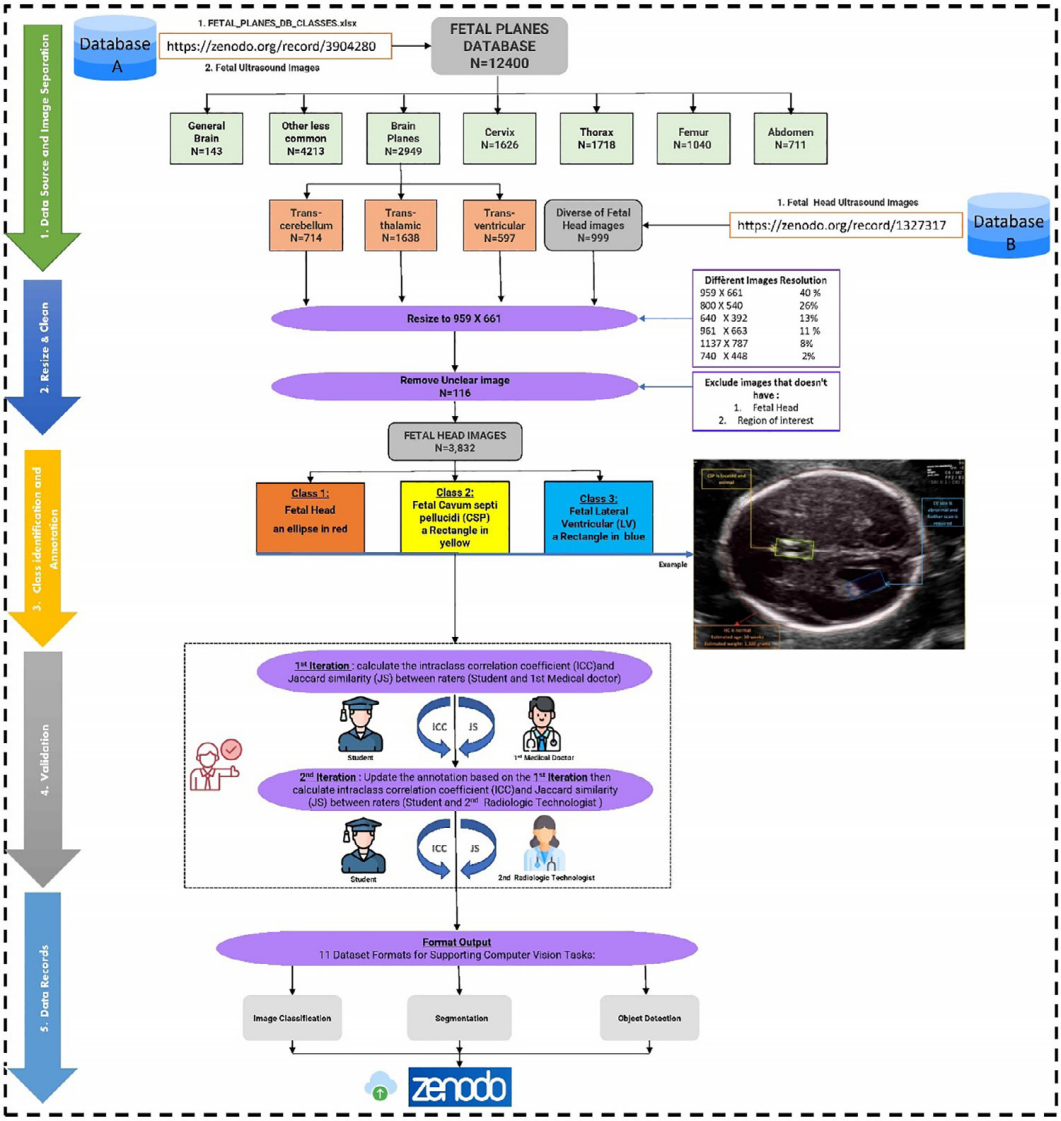
A 5-step workflow from data collection to dataset publication, each detailed in the corresponding guideline for transparency and reproducibility.

In this section, we present the protocol and guidelines that we followed to create a transparent and accurate annotated dataset. Fig. 3 illustrates the entire process, from data collection to dataset publication. Each step shown in (Fig. 3) is elaborated upon in the corresponding guideline, ensuring the transparency and reproducibility of the dataset creation process.

### 3.1 Data source and preprocessing

The ultrasound images integral to this study were obtained from two publicly accessible databases, namely Database A [1] and Database B [2], as illustrated early in Fig. 1. Adhering to the principles of open science, these databases were chosen due to their open availability and compliance with the terms and conditions set forth for data sharing by the data providers.

Database A [1] is a collection of ultrasound images sourced from BCNatal. This center, which consists of two sites (Hospital Clinic and Hospital Sant Joan de Deu, Barcelona, Spain), houses expansive maternal-Fetal departments and facilitates thousands of deliveries annually. The images in database A were collected between October 2018 and April 2019 during standard clinical practices. Pregnant women attending routine second and third trimesters were included in the study, excluding cases of multiple pregnancies, congenital malformations, and aneuploidies. The gestational age (GA), calculated from measurements of the crown rump length (CRL) of the first trimester ultrasound, ranged from 18 to 40 weeks. Despite the wide-ranging images, this database offered a specific subset of images relevant to this study, those showcasing Fetal heads. Hence, a Python script was developed to extract a total of 12,400 images featuring three Fetal head planes (Trans-cerebellum, Trans-Thalamic, and Trans-Ventricular).

Database B [2] is a collection of 1334 two-dimensional (2D) ultrasound head circumference (HC) images. These images were obtained from the Department of Obstetrics of the Radboud University Medical Center, Nijmegen, The Netherlands. The image collection period spanned May 2014 to May 2015, during which routine ultrasound screening exams were performed on 551 pregnant women. The inclusion criteria for this dataset were strictly fetuses with no growth abnormalities. The reference GA was determined using the CRL. The dataset, which contained a mix of images from the head plane of the Fetal, was stratified into a training set and a testing set, accounting for 75 % and 25 % of the images, respectively, with careful balance of the GA. For the purpose of our study, we only utilized the training subset, which comprised 999 images, and reannotated them for further analysis.

In accordance with data sharing conditions, all data sources utilized are openly accessible, ensuring transparency and adherence to relevant licenses. The licensing information for Database A^1^ and Database B^2^ can be accessed using the links provided.

The image resizing process was carried out to standardize the sizes of the images across the dataset, ensuring the preservation of crucial visual information. The images in the dataset, sourced from databases A and B, were found to have varied resolutions. Among these, the resolutions 959 × 661, 800 × 540, 640 × 392, 1137 × 787, and 740 × 448 were predominant, with their aspect ratios ranging between approximately 1.4 to 1.65. Given that the majority of the images were closer to an aspect ratio of 1.45, we opted for a common resolution of 959 × 661 pixels, representing the majority of the dataset (40 %), as illustrated in Fig. 3. Additionally, CNN architectures often require image dimensions that are powers of two for optimal performance [10,11]. Therefore, the choice of this resolution was aimed at making the dataset compatible with various neural network architectures and facilitated smoother processing.

To address any resolution discrepancies, we employed the OpenCV library, utilizing the INTER_AREA interpolation method for downscaling and the INTER_CUBIC method for upscaling, ensuring that the resized images retained high quality. It is essential to emphasize that CNN architectures typically perform resizing and padding automatically to manage images with different aspect ratios. This automatic management ensures that the images are compatible with various neural network architectures without compromising critical visual information. In line with best practices, we also provide the original so that users can adjust/resize masks/labels to match the corresponding to original image size.

Further refinements to the dataset were carried out to enhance clarity and maintain the integrity of the images. By choosing a common resolution that aligned with the majority of the dataset and utilizing specific interpolation methods for downscaling and upscaling, we ensured the preservation of the anatomy's original shape. This approach allowed us to standardize the images without compromising the critical visual information, in line with standard practices in medical imaging.

### 3.2 Annotation tool

We evaluated various open-source tools for image annotation and eventually selected the Computer Vision Annotation Tool (CVAT) as the most suitable for our task. This tool offers various formats for each class, thus optimally addressing our project requirements. Throughout the annotation process, we maintained consistent settings, such as the color and thickness of the annotation lines. In particular, we utilized an ellipse to represent the brain region, which includes the Fetal skull as well as the brain itself, reflecting our emphasis on the overall region of interest. In addition, the presence of the cavum septi pellucidi (CSP) and the lateral ventricle (LV) were denoted by oriented rectangles.

### 3.3 Annotation guidelines

The annotation process involved an individual examination of each plane of the Fetal head (Trans-Thalamic, Trans-Ventricular, Trans-Cerebellum and various head images). The primary objective of our dataset is to focus on the detection and measurement of the brain, the presence of the cavum septi pellucidi (CSP), and the measurement of the lateral ventricle (LV). Consequently, we have employed bounding boxes as masks for segmentation and bounding box coordinates for object detection. We believe that this approach effectively serves our purpose of providing significant information on the presence and measurements of these structures.

To delineate the brain region, we utilized an ellipse, which was subsequently converted into a corresponding mask along with its RLE (run length encoding) [12]. Likewise, rectangles representing the CSP and LV were converted into masks, each encompassing its respective bounding-box coordinates. We discarded any images that did not contain any of these classes.

For each class, a distinct color was used, effectively covering the entire region of interest for that specific class. This approach facilitated precise, accurate, and consistent annotations across all images, with no instances of overlap or underlapping. Importantly, while polygon annotations could potentially offer a more precise delineation of complex boundaries, our use of bounding boxes and ellipses aimed at striking a balance between accuracy, annotation complexity, and the practical utility of the dataset. By simplifying the annotation process, we have ensured our dataset's accessibility and usability for a wider range of applications and researchers, despite the relatively lower structural fidelity of bounding boxes and ellipses compared to richer semantic labels.

### 3.4 Annotation procedure

We followed a structured procedure to complete the annotation process, which involved several steps:

1. The Ph.D. student (First Author) attended five teaching sessions with a Senior Attending Physician (Third Author), who had 12 years of experience in maternal-Fetal medicine, in a medical center to improve their understanding of the structures, characteristics, and categories relevant to identifying structures in ultrasound images.
2. Ph.D. student performed the initial annotation using CVAT by drawing boundaries around each labeled structure, ensuring that the boundaries accurately reflected the extent of the structure in the Fetal head ultrasound image.
3. The Senior Attending Physician specializing in Maternal-Fetal Medicine reviewed the annotations provided by the Ph.D. Student on a class-by-class basis, offering feedback as necessary.
4. Ph.D. student revised the annotations based on feedback from the senior attending physician.
5. An experienced Radiologic Technologist (Fourth Author) with expertise in Obstetric/Gynecologic Sonography, who had 10 years of experience, reviewed the final version of the annotations prepared by the Ph.D. student. This review involved randomly sampling images and providing feedback.
6. The Ph.D. student further revised the annotations on the basis of the feedback provided by the Radiologic Technologist and subsequently prepared the final dataset.

### 3.5 Validation

Our validation process, essential to the robustness and comprehensiveness of our dataset, incorporated the Intraclass Correlation Coefficient (ICC) [13] and Jaccard similarity (JS) [14] measures. These metrics provided a thorough evaluation of the agreement and similarity between the annotators.

The ICC, an effective measure of interrater reliability, evaluated the agreement between annotators in terms of the binary correctness of the class locations. When the class locations annotated by the PhD student were confirmed as accurate by the Senior Attending Physician (SAP) or Radiologic Technologist (RT), a value of 1 was assigned. On the contrary, incorrect locations received a value of 0, and corrective instructions were provided. Therefore, the ICC was beneficial in gauging the reliability of the annotations.

The JS measure, used to quantify the proportion of shared elements between two sets, allowed us to evaluate the overlap of correct annotations made by the reviewers. This metric was particularly suited for our binary correctness data, providing insight into the consistency of the annotations.

The validation process, illustrated in (Fig. 3), consisted of two iterations of rigorous examination and refinement. Both SAP and RT independently assessed the annotations without access to each other's feedback or comments. This protected the process from any potential bias, enhancing the reliability of the dataset.

• **1st Iteration**
  ○ The PhD student annotated all images, which were subsequently examined by the SAP. Discrepancies were identified and corrective instructions were provided. The inter-rater reliability was then evaluated using both ICC and JS, assessing the agreement between the PhD student and the SAP.
  ○ The PhD student incorporated the SAP's feedback, refining the annotations for all images and classes accordingly.
• **2nd Iteration**
  ○ The RT reviewed a random sample of images, constituting 20 % of the dataset, and offered feedback. The inter-rater reliability was assessed once again using both ICC and JS, gauging the concordance between the PhD student and the RT's annotations.
  ○ Based on the RT's feedback, the PhD student adjusted the annotations for all images and classes where required.

The validation and interrater reliability assessment of the annotated dataset was achieved through a robust two-step process. Initially, a Ph.D. student annotated all classes for each Fetal plane group. The Senior Attending Physician subsequently reviewed these annotations. The intra-class correlation coefficient (ICC (2,1)) and Jaccard similarity (JS) were used to quantitatively measure the level of agreement and similarity between the annotations made by the student and the physician for each class in each group. The calculation of ICC (2,1) and JS is represented in Eqs. 1 and 2:

(1) $$ICC\left(2,1\right)=\frac{MS_{between}-MS_{within}}{MS_{between}+\left(k-1\right)MS_{within}}$$

where $MS_{between}$ refers to the mean square between groups, $MS_{within}$ represents the mean square within groups, and k is the number of raters.

(2) $$JS\left(A,B\right)=\frac{\left|A\cap B\right|}{\left|A\cup B\right|}$$

where A and B are the sets of labels assigned by the two annotators, |A∩B|represents the size of the intersection of sets A and B, and |A∪B|represents the size of the union of sets A and B.

Values indicating the level of agreement (that is, reliability between the parties) between the student and the physician (1st iteration), and the student and a radiologic technologist (2nd iteration) are systematically organized by Fetal plane groups and presented in Table 2. Higher ICC and JS values are indicative of better agreement between the two annotators.

**Table 2:** Intraclass Correlation Coefficient (ICC) and Jaccard similarity (JS) values for inter-rater reliability by fetal plane.

1st iteration: Rater Reliability between Student and Physician							
Fetal Plane Group	Number of images	Brain ICC	CSP ICC	LV ICC	Brain JS	CSP JS	LV JS
Trans-Thalamic	1565	0.940	0.939	0.662	0.999	0.929	0.603
Trans-Ventricular	584	1.00	0.985	0.974	1.00	0.989	0.989
Trans-Cerebellum	684	1.00	0.792	0.218	1.00	0.818	0.125
Diverse head images	999	1.00	0.871	0.926	1.00	0.875	0.930

2nd iteration: Rater Reliability between Student and Radiologic Technologist							
Trans-Thalamic	301	1.00	0.853	0.80	1.00	0.760	0.67
Trans-Ventricular	110	1.00	0.854	0.855	1.00	0.878	0.958
Trans-Cerebellum	150	1.00	0.865	0.662	1.00	0.833	0.50
Diverse head images	200	1.00	0.887	0.892	1.00	0.840	0.849

In the 1st iteration (Student and Physician), the agreement on brain annotation is excellent, with ICC and JS values consistently at 1 in all groups. The agreement on CSP annotation is generally good, with the highest agreement in the transventricular plane (ICC = 0.985, JS = 0.989) and the lowest in the transcerebellum plane (ICC = 0.792, JS = 0.818). The agreement on the annotation of the LV varies, with a high agreement in the transventricular plane (ICC = 0.974, JS = 0.989) and a low agreement in the transcerebellum plane (ICC = 0.218, JS = 0.125). The overall average ICC for the first iteration is approximately 0.859, calculated as (0.847 + 0.986 + 0.670 + 0.932)/4. Similarly, the overall average JS for the first iteration is approximately 0.855, calculated as (0.844 + 0.993 + 0.648 + 0.935)/4.

In the 2nd iteration (Student and Radiologic Technologist), the agreement on brain annotation is excellent, with ICC and JS values consistently at 1 in all groups. The agreement on CSP annotation is also generally good but slightly lower than in the first iteration. The highest agreement is in the diverse head images group (ICC = 0.887, JS = 0.840), and the lowest is in the transthalamic plane (ICC = 0.853, JS = 0.760). The agreement on LV annotation varies again, with the highest agreement in the transventricular plane (ICC = 0.855, JS = 0.958) and the lowest in the transcerebellum plane (ICC = 0.662, JS = 0.5). The overall average ICC for the second iteration is approximately 0.889, calculated as (0.884 + 0.903 + 0.842 + 0.926)/4. Similarly, the overall average JS for the second iteration is approximately 0.857, calculated as (0.810 + 0.945 + 0.778 + 0.896)/4.

The relatively lower agreement on LV annotation in the transcerebellum plane could be attributed to inherent difficulties in visualizing the LV in this plane and the fewer instances of LV class identified. In the context of the diverse head images, a difference of opinion emerged between the Radiologic Technologist, who advocated for the exclusion of these images, and the Senior Physician, who argued for their inclusion, particularly because they were taken at an early gestational age below 14 weeks. The decision to include these images, guided by the Senior Physician's recommendation, underscores the importance of expert involvement and inter-rater reliability assessment in developing a robust, accurate dataset for machine learning models.

## 4 Limitations

In this dataset, two key limitations warrant mention. First, the dataset lacks the DICOM (Digital Imaging and Communications in Medicine) format, a standard extensively used in commercial medical imaging. While the dataset supports 11 different universally accepted formats compatible with various software like ImageJ, 3D Slicer, and ITK-SNAP, this absence may constrain researchers and clinicians reliant on DICOM-based systems. Our dataset is also optimized for open-source deep learning frameworks, potentially not meeting the specifications of those using proprietary software. Second, to enhance compatibility with neural network architectures, we resized the images to a common resolution. This resizing facilitates smoother data processing but may compromise the finer details critical to some analyses. To counteract this, we included the original, pre-resized images in the repository. However, using these originals necessitates handling variable image sizes and resolutions, adding a layer of complexity. Thus, the resizing introduces a trade-off between broad usability and the preservation of original image details.

## Ethics Statement

This study employed publicly available Fetal ultrasound images obtained under the Creative Commons Attribution 4.0 International (CC BY 4.0) license. All images used were previously anonymized and de-identified to protect patient confidentiality and privacy. Our dataset creation followed the guidelines stipulated by the CC BY 4.0 license, including proper attribution to the original author(s) and indication of any modifications made. This new dataset was also published under the same CC BY 4.0 license, in compliance with the terms of use, adaptation, and redistribution. The ethical requirements outlined by the CC BY 4.0 license were thoroughly reviewed to ensure complete compliance.

Furthermore, for the sake of transparency, although our work does not require ethical approval, we obtained an exempt review status from the Hamad Bin Khalifa University - Institutional Review Board (HBKU-IRB). The IRB Protocol Reference Number is QBRI-IRB-2023-24, and the project title is “A Comparative Analysis of Deep Learning and Sonographer Evaluations in Fetal Ultrasound Imaging: An Intraclass Correlation Study.” The review type is listed as Exempt, confirming that our project adheres to ethical standards in research.

This statement confirms that we have read and followed the ethical requirements for publication in Data in Brief and have taken additional steps to ensure the ethical integrity of our dataset.

## CRediT authorship contribution statement

**Mahmood Alzubaidi:** Data curation, Formal analysis, Writing – original draft. **Marco Agus:** Data curation, Writing – review & editing. **Michel Makhlouf:** Data curation, Validation. **Fatima Anver:** Validation. **Khalid Alyafei:** Supervision. **Mowafa Househ:** Supervision.

## Data Availability

Large-Scale Annotation Dataset for Fetal Head Biometry in Ultrasound Images (Original data) (Zenodo).

## Footnotes

1 https://zenodo.org/record/3904280.

2 https://zenodo.org/record/1322001.
